# A Hypothesis From Metabolomics Analysis of Diabetic Retinopathy: Arginine-Creatine Metabolic Pathway May Be a New Treatment Strategy for Diabetic Retinopathy

**DOI:** 10.3389/fendo.2022.858012

**Published:** 2022-03-24

**Authors:** Ye Sun, Ling Kong, Ai-Hua Zhang, Ying Han, Hui Sun, Guang-Li Yan, Xi-Jun Wang

**Affiliations:** ^1^National Chinmedomics Research Center and National Traditional Chinese Medicine (TCM) Key Laboratory of Serum Pharmacochemistry, Department of Pharmaceutical Analysis, Heilongjiang University of Chinese Medicine, Harbin, China; ^2^State Key Laboratory of Quality Research in Chinese Medicine, Macau University of Science and Technology, Macau, Macau SAR, China; ^3^National Engineering Laboratory for the Development of Southwestern Endangered Medicinal Materials, Guangxi Botanical Garden of Medicinal Plant, Nanning, China

**Keywords:** diabetic retinopathy, metabolomics, biomarker, creatine, arginine, mechanism

## Abstract

Diabetic retinopathy is one of the serious complications of diabetes, which the leading causes of blindness worldwide, and its irreversibility renders the existing treatment methods unsatisfactory. Early detection and timely intervention can effectively reduce the damage caused by diabetic retinopathy. Metabolomics is a branch of systems biology and a powerful tool for studying pathophysiological processes, which can help identify the characteristic metabolic changes marking the progression of diabetic retinopathy, discover potential biomarkers to inform clinical diagnosis and treatment. This review provides an update on the known metabolomics biomarkers of diabetic retinopathy. Through comprehensive analysis of biomarkers, we found that the arginine biosynthesis is closely related to diabetic retinopathy. Meanwhile, creatine, a metabolite with arginine as a precursor, has attracted our attention due to its important correlation with diabetic retinopathy. We discuss the possibility of the arginine-creatine metabolic pathway as a therapeutic strategy for diabetic retinopathy.

## Introduction

Diabetic retinopathy (DR) has been recognized as the main cause of blindness worldwide, with about one-third of all diabetes patients developing diabetic retinopathy (1). The retina is metabolically active and transmits electrochemical signals from photoreceptors to the brain *via* neurons, supported by glial cells and vascular tissue (2). The entire process relies on highly complex coordination between the various cell types, and the blood-vision barrier plays a key role (3, 4). The accumulation of glycation end products, oxidative stress, polyol pathway and protein kinase C (PKC) activation are the main pathogenesis of DR. This changes the normal interaction between cells and causes serious blood vessel abnormalities leading to damaging of the blood-retinal barrier and neuronal function (5–9). Diabetic retinopathy is difficult to cure, diagnosis and drug intervention in the early stages of diabetic retinopathy can effectively prevent or slow down the progression of disease. Therefore, identification of biomarkers associated with disease progression can be very helpful.

Metabolomics is the analysis of a large number of endogenous small molecules. It provides the overall metabolic profile of a biological sample as opposed to genomics and proteomics, which provide the profiles for DNA/RNA and proteins alone, respectively (10–12). The methods of analysis used in metabolomics are mostly classified into two categories: targeted metabolomics and non-targeted metabolomics (13–15). In contrast to targeted metabolomics, which focuses only on changes in specific metabolites, non-targeted metabolomics is designed to capture much more metabolite information to compare these high-throughput data under normal vs. disease states (15–17). Non-targeted metabolomics approaches can thus discover potential biomarkers of diseases and provide an effective basis for diagnosing and treating them (18–20).

Arginine, a semi-essential amino acid, involved in many biological processes such as creatine biosynthesis and the urea cycle, is one of the strongest insulin secretagogues, which induce insulin release from pancreatic β cells (21). Additionally, arginine is a substrate for nitric oxide synthase (NOS) and can produce NO, which exerts a significant influence on the health of the vascular endothelial cells as well as the kidneys (22, 23). Creatine (Cr) can be either be synthesized endogenously within the body or extrinsically derived from foods like meat, fish, etc. (24). Cr, phosphocreatine(PCr), and creatine kinase (CK) isoenzymes are responsible for maintaining the ATP pool (25). Therefore, creatine is one of the leading sports supplements (26). As research continues, Creatine has been found to have multiple physiological effects, including anti-inflammatory (27–29), antioxidant (30–33), neuroprotective (34), reduce homocysteine(Hcy) (35–37), and anti-diabetic (34).

This review aims to summarize the progress of metabolomics studies in diabetic retinopathy and to explore common research platforms for metabolomics. We also summarize the current knowledge of known metabolomics biomarkers of diabetic retinopathy based on literature and analyze the metabolic pathways involving those biomarkers. In addition, we discuss the creatine-arginine metabolic network as a potential area for finding new treatment strategies.

## Metabolomics Analysis Platform

Metabolomics analysis platform can be divided into two main types, nuclear magnetic resonance (NMR) spectroscopy (38) and mass spectrometry (MS) (39, 40). Using different instruments and platforms, typically 50 to as many as 5000 different metabolites can be identified at any given time. No technique so far has been successful in identifying all metabolites in a single run or analysis, and most metabolomics studies use only one platform or multiple tandems. Due to the complementarity between NMR (41) and MS (42), researchers often use combinations of NMR and MS as well as employ the current method to enhance research quality and expand the metabolome coverage (43–46).

### Nuclear Magnetic Resonance (NMR) Spectroscopy

NMR spectroscopy can measure the behavior of an atom’s nucleus when subjected to a magnetic field (47, 48). Currently, instruments that use 500 and 600 MHz frequencies are the most widely used instruments to detect these signals and are the optimal choice for their sensitivity and manufacturing cost. It is worth noting that the resolution of these signals increases when the magnetic field strength is higher (49).

NMR spectroscopy applies to both liquid/gas phase samples as well as tissue samples (50, 51). It carries several advantages, for example, it requires less sample preparation and the detection process is non-destructive to the sample, so it can be reused for other studies. Moreover, NMR has high reproducibility and good quantitative performance, allowing the measurement of the number of protons under a given condition which allows for direct comparison with spectral data (52). However, the primary disadvantage of NMR is its lower sensitivity compared with MS. NMR can identify nearly 50 metabolites in serum/plasma samples and approximately 200 in urine (53).

### Mass Spectrometry (MS)

Mass spectrometry is an analytical method that measures the ion-to-mass ratio based on the ionization of components in the samples by an ion source, and is widely used in the detection of metabolites (54–56). The sample can be directly analyzed by mass spectrometry, or in tandem with other separation methods to obtain mass spectra, such as liquid chromatography (LC) (57–59), gas chromatography (GC) (60, 61), hydrophilic interaction chromatography-mass spectrometry (HILIC-MS) (62), Flow-injection analysis-mass spectrometry(FIA–MS) (63), or capillary electrophoresis (CE) (64, 65). It should be noted that no single method can separate all metabolites simultaneously, as some metabolites are difficult to ionize, and in some cases, mass number limitations prevent mass spectrometry techniques from measuring all metabolites (66). LC has been most widely used because of its better separation. Especially, high-performance liquid chromatography (HPLC) and ultra-high performance liquid chromatography (UPLC) have become increasingly popular (67–69). GC also offers high separation, but it is unable to measure metabolites with poor thermal stability (70). Capillary electrophoresis (CE) has a long history of use. Its application is mainly limited by its poor sensitivity, which has been greatly improved by the introduction of the CE-ESI interface (71, 72).

Compared to NMR, MS has a much higher sensitivity and is therefore able to measure a wider range of metabolites (40, 43, 73, 74). In particular, UPLC offers excellent chromatographic separation, high speed, and high sensitivity, allowing the detection of thousands of metabolites within a short time (75–78). HPLC tandem MS plays a huge contribution in research that requires high throughput, such as natural drug development and disease biomarker identification (79–83).

## Biomarkers for Diabetic Retinopathy

### Vitreous Humor Biomarkers

Tomita et al. (84) analyzed the metabolites of vitreous humor in 43 proliferative diabetic retinopathy (PDR) patients, and 21 controls using ultra-performance liquid chromatography-mass spectrometry (UPLC-MS) with significant differences in creatine. The authors found that patients with PDR had lower levels of creatine and higher levels of glycine in the vitreous humor than controls. They also verified in an oxygen induced ischemic retinopathy (OIR) model that reduced creatine levels correlate with retinal vascular proliferation and demonstrated that oral creatine caused a significant reduction in retinal vascular proliferation (p=0.0024), opening the possibility for a new therapeutic strategy for diabetic retinopathy. Wang et al. (85) identified potential DR biomarkers in vitreous humor using gas chromatography mass spectrometry (GC-MS). Vitreous humor samples were gathered from 28 type-2 diabetes patients with PDR as well as 22 non-diabetic patients with macular fissure. They found 15 potential biomarkers in the vitreous humor, namely pyruvate, ornithine, uric acid, pyroglutamic acid, creatinine, L-leucine, L-alanine, L-threonine, L lysine, L-valine, L-phenylalanine, L-isoleucine, L-glutamine, inositol, and hydroxylamine. These are mainly involved in various metabolic pathways such as gluconeogenesis, ascorbate-aldose metabolism, valine-leucine-isoleucine biosynthesis, and arginine-proline metabolism.

A non-targeted metabolomics study on vitreous humor from patients with DR showed changes in glucose metabolism as well as activation of the pentose phosphate pathway. Glass fluid samples from PDR patients (n=9) and normal subjects were kept as controls (n=8) and were analyzed by ultra-performance liquid chromatography-mass spectrometry (UPLC-MS). A variety of metabolites were found to be potential biomarkers, including xanthine, pyruvate, proline, and guanine (86). Paris et al. (62) used liquid chromatography-mass spectrometry (LC-MS) and hydrophilic interaction liquid chromatography (HILIC)-mass spectrometry to analyze the vitreous humor of PDR patients (n=9), non-diabetes control patients (n=11), and OIR mouse model. They found significant changes in the levels of octanoyl carnitine, propionyl carnitine, hexanoyl carnitine, acetylcarnitine, palmitoylcarnitine, elaidic/vaccenylcarnitine, allantoin, glutamate, lysine, and arginine. Barba et al. (87) analyzed the vitreous humor of a total of 22 patients suffering from PDR and 22 non-diabetic patients and found that the content of lactate and glucose among the PDR patients was higher than that in non-diabetic patients, while that of galactitol and ascorbic acid was lower when compared with that in non-diabetic patients. The reduced galactitol level was attributed to activation of the polyol pathway.

### Plasma Biomarkers

Plasma metabolomics of 124 DR patients and 32 controls were explored using GC–MS, and UPLC–MS. They identified glutamine and glutamic acid as new biomarkers for the prediction of DR (88). A plasma metabolomics analysis based on GC–MS demonstrated that 2,4-dihydroxybutyric acid (DHBA), 3,4-DHBA, ribonic acid, and ribitol are risk markers for DR progression as these metabolites are associated (P <0.042) with DR (89). Another plasma metabolomics study using GC-MS identified 11 potential biomarkers of diabetic retinopathy, namely 1,5-gluconolactone, 1,5-anhydroglucitol, gluconic acid, lactose/cellobiose, maltose/trehalose, 2-deoxyribonic acid, 3,4-dihydroxybutyric acid, erythritol, mannose, ribose, and urea. The samples for this study were acquired from 40 patients undergoing non-proliferative diabetic retinopathy (NPDR) and 40 patients suffering from T2DM without retinopathy. Metabolic pathway analysis indicated a remarkable enrichment of the pentose phosphate pathway, which could explain the NADPH production against oxidative stress (49). Sumarriva et al. performed plasma metabolomics research showed that compared to diabetes controls, the metabolism of multiple amino acids, such as leukotrienes, niacin, pyrimidine, and purine, changed in DR patients. Arginine, citrulline, glutamic γ-semialdehyde, and de-hydroxy carnitine were critical members in the above pathways differences (90). Li et al. (91) employed GC-MS in the study of plasma metabolomics in 25 patients with PDR, 39 patients with NPDR, and 24 patients with NDR, and found 10 metabolites with significant differences: β-hydroxybutyrate, methylmalonic acid, citric acid, pyruvate, glucose, stearic acid trans-oleic acid, L-aspartate, linoleic acid, and arachidonic acid.

### Serum Biomarkers

Xuan et al. (92) studied 43 patients with diabetic retinopathy and 44 normally controlled serum lipomics using UPLC-MS. Significant differences were found in the following 14 lipid metabolites: Lysophosphatidylcholine(LPC)(14:0) LPC (14:0), LPC (16:0) LPC (14:0), LPC (16:0), LPC (16:1), LPC (18:0), LPC (18:1), LPC (18:2), LPC (18:3), LPC (18:4), LPC (20:0), LPC (20:3), LPC (20:4), LPC (20:5), LPC (22:3), and LPC (22:6). These provide a basis for the discovery of lipid biomarkers in diabetic retinopathy. Xuan et al. (93) in their study used multi-platform techniques to analyze serum samples from 111 diabetic patients without retinopathy (NDR=111) and 350 diabetic patients with retinopathy (n=350). The DR-induced metabolic changes were usually linked to glycolytic metabolism, tricarboxylic acid cycle (TCA) metabolism, urea cycle metabolism, polyol metabolism, amino acid metabolism, and lipid metabolism. Following a systematic screening using univariate analysis, 2-piperidone and 12-HETE were recognized as potential biomarkers for DR. 12-HETE, an eicosanoid-like acid, is the leading product of human 12-lipoxygenase (LOX), inducing endoplasmic reticulum stress in human retinal endothelial cells. Studies show that 12-LOX is involved in retinal microvascular disorders of DR (94–96). A study based on widely targeted metabolomics evaluated serum metabolites from 69 type 2 diabetes mellitus (T2DM) patients with DR and 69 T2DM patients without DR. The biomarkers of diabetic retinopathy identified using a UPLC-MS system were linoleic acid, nicotinuric acid, ornithine, and phenylacetylglutamine. In particular, this research developed a new multidimensional network of biomarker systems and the area under the curve (95% CI) of this system is an exploration of the biomarker determination method (97).

Zhu et al. (98) studied the serum metabolomics of 21 PDR patients and 21 diabetic patients without retinopathy (NDR) patients. A total of 63 significant changes in metabolites were found using LC-MS. Fumaric acid, uridine, acetic acid, and cytidine (area under curve 0.96, 0.95, 1.0, and 0.95, respectively) are considered potential biomarkers of PDR. A serum metabolomics study of 24 patients with PDR, 22 patients with NPDR, and 35 healthy human control groups demonstrated that compared with the control group, indolamine-2,3-dioxygenase (IDO) expression was enhanced among patients with NPDR, while the levels of kynurenine, kynurenic acid, and 3-hydroxy kynurenine were higher in PDR patients. The authors speculated that diabetic retinopathy might be related to IDO and tryptophan metabolites (99). Serum samples from patients with NPDR (n=123), PDR (n=51), and NDR (n=143) were profiled by targeted mass-spectrometry-based metabolomics. After multivariate analyses, 16 metabolites were found to show profound changes, including tetradecenoylcarnitine (C14:1), hexadecanoylcarnitine (C16), lysine, methionine, tryptophan, tyrosine, total dimethyarginine, phosphatidylcholine diacyl C32:2, phosphatidylcholine diacyl C34:2, phosphatidylcholine diacyl C36:2, phosphatidylcholine diacyl C38:6, phosphatidylcholine diacyl C40:6, phosphatidylcholine acyl-alkyl C36:5, phosphatidylcholine acyl-alkyl C42:3, hydroxysphingomyeline C22:1 and sphingomyeline C24:0 (63).

### Aqueous Humor Biomarkers

Wang et al. (85) analyzed and identified potential DR biomarkers in aqueous humor of 23 patients suffering from PDR and 25 patients with non-diabetic cataracts. Eight metabolites, namely D-glyceric acid, isocitric acid, threonine, d-glucose, inositol, L-lactic acid, citrulline, and fructose 6-phosphate, were found to be significantly different in the aqueous humor by comparative analysis.

A metabolomics study based on NMR was carried out on the aqueous humor samples from diabetic patients with cataracts (n=13), DR patients with cataracts (n=14), and elderly cataracts (n=7). Metabolites such as lactate, succinate, 2-hydroxybutyrate, aspartamide, dimethylamine, histidine, threonine, and glutamine showed significant changes. Pathway analysis showed that DR might be related to alanine, aspartic acid, and glutamate metabolic pathways (100). The information of DR biomarker was listed in **Table 1**.

## KEGG Enrichment Analysis

**Figure 1:**
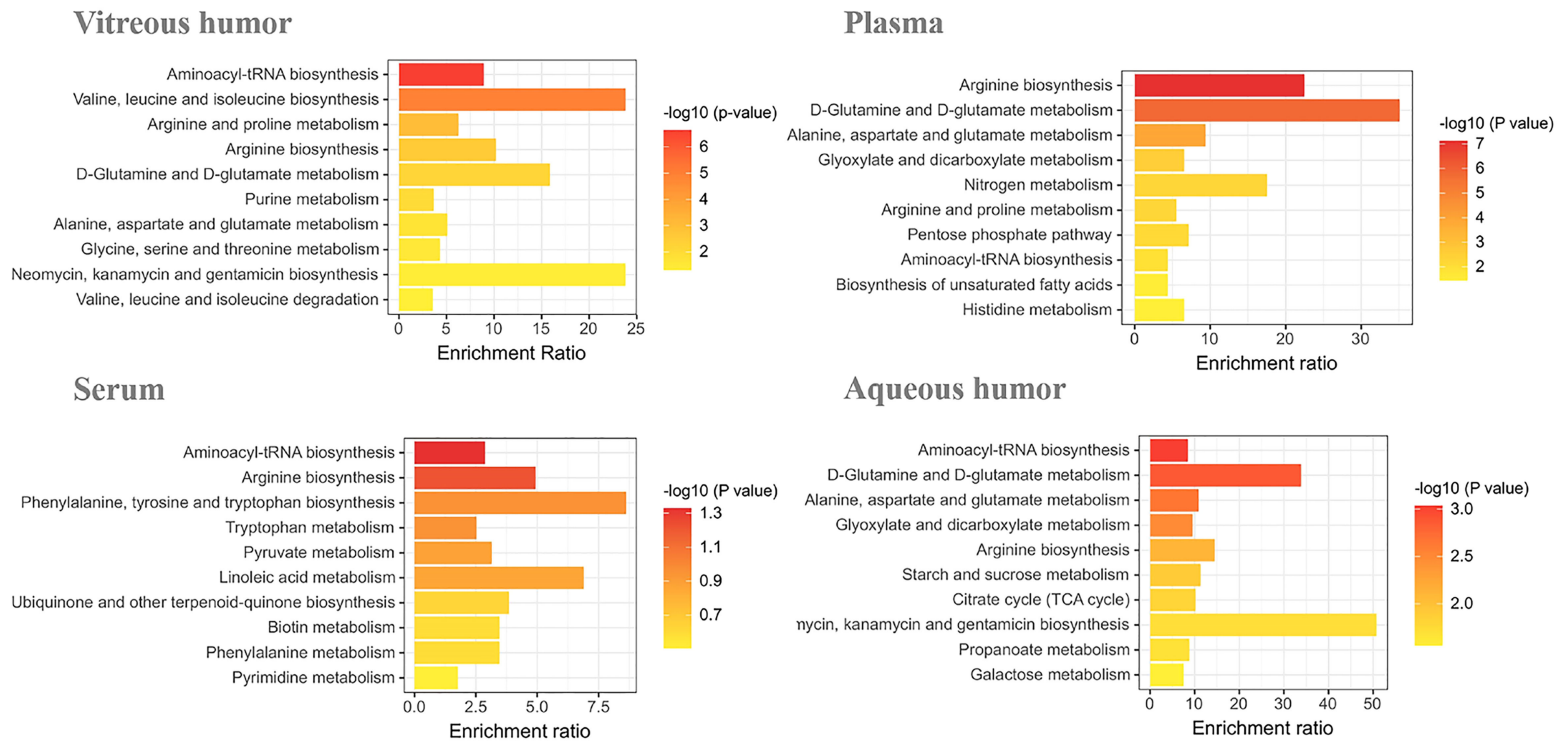
Enrichment analysis of DR potential biomarkers in vitreous humor, plasma, serum, and aqueous humor.

We enriched the above potential biomarkers according to the types of biological fluids, intending to comprehend the relationship between biomarkers and diseases. Enrichment analysis by metaPA and Kyoto Encyclopedia of Genes and Genomes (KEGG) showed that metabolic pathways enriched in the different biological fluids are unique (**Figure 1**). It is worth mentioning that arginine-related metabolism was both enriched in vitreous humor, plasma, serum, and aqueous humor. This suggests that arginine has a critical effect on diabetic retinopathy.

## Discussion

Biomarkers can provide early warning signs in patients with serious diseases. Therefore, they help in the early diagnosis of the disease so that effective treatment can be made available to the patient at the earliest. In this review, we have summarized the known potential biomarkers for DR, in a variety of biological samples, including vitreous humor, plasma, serum, and aqueous humor, from research done in recent years. Through enrichment analysis, we found that arginine-related metabolic pathways were abnormal in a variety of biological fluids.

**Table 1:** The information of diabetic retinopathy biomarker.

Study	Samples	Platform	Number (cases/model and controls)	Potential biomarkers	Pathways
Tomita et al. (84)	Vitreous humour	UPLC-MS	43 PDR and 21 non-diabetic epiretinal membrane	Creatine, succinate, glycine, lactate, pyruvate, proline, allantoin, urate, citrulline, ornithine, dimethylglycine, N-acetylserine, α-ketoglutarate	Glycine, serine, arginine and proline amino acid metabolism
Wang et al. (85)	Vitreous humour	GC-TOF-MS	28 PDR and 22 non-diabetic patients with macular fissure	Pyruvate, ornithine, uric acid, pyroglutamic acid, creatinine, L-leucine, L-alanine, L-threonine, L lysine, L-valine, L-phenylalanine, L-isoleucine, L-glutamine, inositol, and hydroxylamine	Gluconeogenesis, ascorbate-aldose metabolism, valine-leucine-isoleucine biosynthesis, and arginine-proline metabolism
Haines et al. (86)	Vitreous humour	UPLC-MS	9 PDR and 8 non-diabetic patients	Xanthine, pyruvate, proline, and guanine	Unclear
Paris et al. (62)	Vitreous humour	LC-MS and HILIC-MS	9 PDR and 11 non-diabetic patients	Octanoylcarnitine, propionylcarnitine, hexanoylcarnitine, acetylcarnitine, palmitoylcarnitine, elaidic/vaccenylcarnitine, allantoin, glutamate, lysine, and arginine	Unclear
Barba et al. (87)	Vitreous humour	NMR	22 PDR and 22 non-diabetic patients	Lactic acid, glucose, galactitol, and ascorbic acid	Unclear
Rhee et al. (88)	Plasma	GC–TOF–MS and UPLC–Q–TOF–MS	124 DR and 32 NDR	Glutamine and glutamic acid	Unclear
Curovic et al. (89)	Plasma	GC-MS	141 DR and 504 NDR	2,4-dihydroxybutyric acid (DHBA), 3,4-DHBA, ribonic acid, and ribitol	Unclear
Chen et al. (49)	Plasma	GC-MS	44 NPDR and 40 NDR	1,5-Anhydroglucitol, 1,5-gluconolactone, 2-deoxyribonic acid, 3,4-dihydroxybutyric acid, erythritol, gluconic acid, lactose/cellobiose, maltose/trehalose, mannose, ribose, and urea	Pentose phosphate pathway
Sumarriva et al. (90)	Plasma	LC-MS	83 DR and 90 NDR	Arginine, citrulline, glutamic γ-semialdehyde, and dehydroxycarnitine	The metabolism of multiple amino acids, leukotrienes, niacin, pyrimidine, and purine
Li et al. (91)	Plasma	GC-MS	25 PDR, 39 NPDR, and 24 NDR	Pyruvate, L-aspartate, β-hydroxybutyrate, methylmalonic acid, citric acid, glucose, stearic acid trans-oleic acid, linoleic acid, and arachidonic acid	Unclear
Xuan et al. (92)	Serum	UPLC - MS	44 PDR and 43 non-diabetic patients	LPC (14:0), LP (16:0), LPC (14:0), LPC (16:0), LPC (16:1), LPC (18:0), LPC (18:1), LPC (18:2), LPC (18:3), LPC (18:4), LPC (20:0), LPC (20:3), LPC (20:4), LPC (20:5), LPC (22:3), and LPC (22:6)	Unclear
Xuan et al. (93)	Serum	GC-MS, LC-MS	350 DR and 111 NDR	2-Piperidone and 12-HETE	Unclear
Zuo et al. (97)	Serum	UPLC-MS	69 DR and 69 NDR	Linoleic acid, nicotinuric acid, ornithine, and phenylacetylglutamine	Unclear
Zhu et al. (98)	Serum	LC-MS	44 NPDR and 40 NDR	Fumaric acid, uridine, acetic acid, and cytidine	Alanine, aspartate and glutamate metabolism, caffeine metabolism, beta-alanine metabolism, purine metabolism, cysteine and methionine metabolism, sulfur metabolism, sphingosine metabolism, and arginine and proline metabolism
Munipally et al. (99)	Serum	HPLC	24 PDR, 22 NPDR, and 35 healthy human control group	kynurenine, kynurenic acid, and 3-hydroxy kynurenine	Tryptophan metabolites
Yun et al. (63)	Serum	LC-MS and FIA-MS	123 NPDR, 51 PDR, and 143 NDR	Tetradecenoylcarnitine, hexadecanoylcarnitine, lysine, methionine, tryptophan, tyrosine, total Dimethyarginine, phosphatidylcholine diacyl C32:2, phosphatidylcholine diacyl C34:2, phosphatidylcholine diacyl C36:2, phosphatidylcholine diacyl C38:6, phosphatidylcholine diacyl C40:6, phosphatidylcholine acyl-alkyl C36:5, phosphatidylcholine acyl-alkyl C42:3,hydroxysphingomyeline C22:1, and phingomyeline C24:0	Unclear
Wang et al. (85)	Aqueous humor	GC-TOF-MS	23 PDR and 25 NDR	D-glyceric acid, isocitric acid, threonine, d-glucose, inositol, L-lactic acid, citrulline, and fructose 6-phosphate	Unclear
Jin et al. (100)	Aqueous humor	NMR	13 diabetic patients with cataract, 14 DR with cataract, and 7 elderly cataract	Lactate, succinate, 2-hydroxybutyrate, aspartamide, dimethylamine, histidine, threonine, and glutamine	Alanine, aspartic acid and glutamate metabolic pathways

LC-MS, liquid chromatography-mass spectrometry; HPLC, ultra-performance liquid chromatography; UPLC-MS, ultra-performance liquid chromatography-mass spectrometry; UPLC–Q–TOF–MS, ultra-performance liquid chromatography quadrupole time-of-flight mass spectrometry; GC-MS, gas chromatography mass spectrometry; GC-TOF-MS, gas chromatography quadrupole time-of-fight mass spectrometry; HILIC-MS hydrophilic interaction chromatography-mass spectrometry; NMR, nuclear magnetic resonance; FIA–MS, flow-injection analysis-mass spectrometry; UPLC-Q-Axis Orbiter-MS, ultra-performance liquid chromatography-quadrupole-Exactive Orbitrap-mass spectrometry; DR, diabetic retinopathy; NDR, diabetic patients without retinopathy; PDR, proliferative diabetic retinopathy; NPDR, non-proliferative diabetic retinopathy; LPC, Lysophosphatidylcholine.

### Arginine Biosynthesis-Related Metabolites Are Significantly Elevated in DR Patients

**Figure 2:**
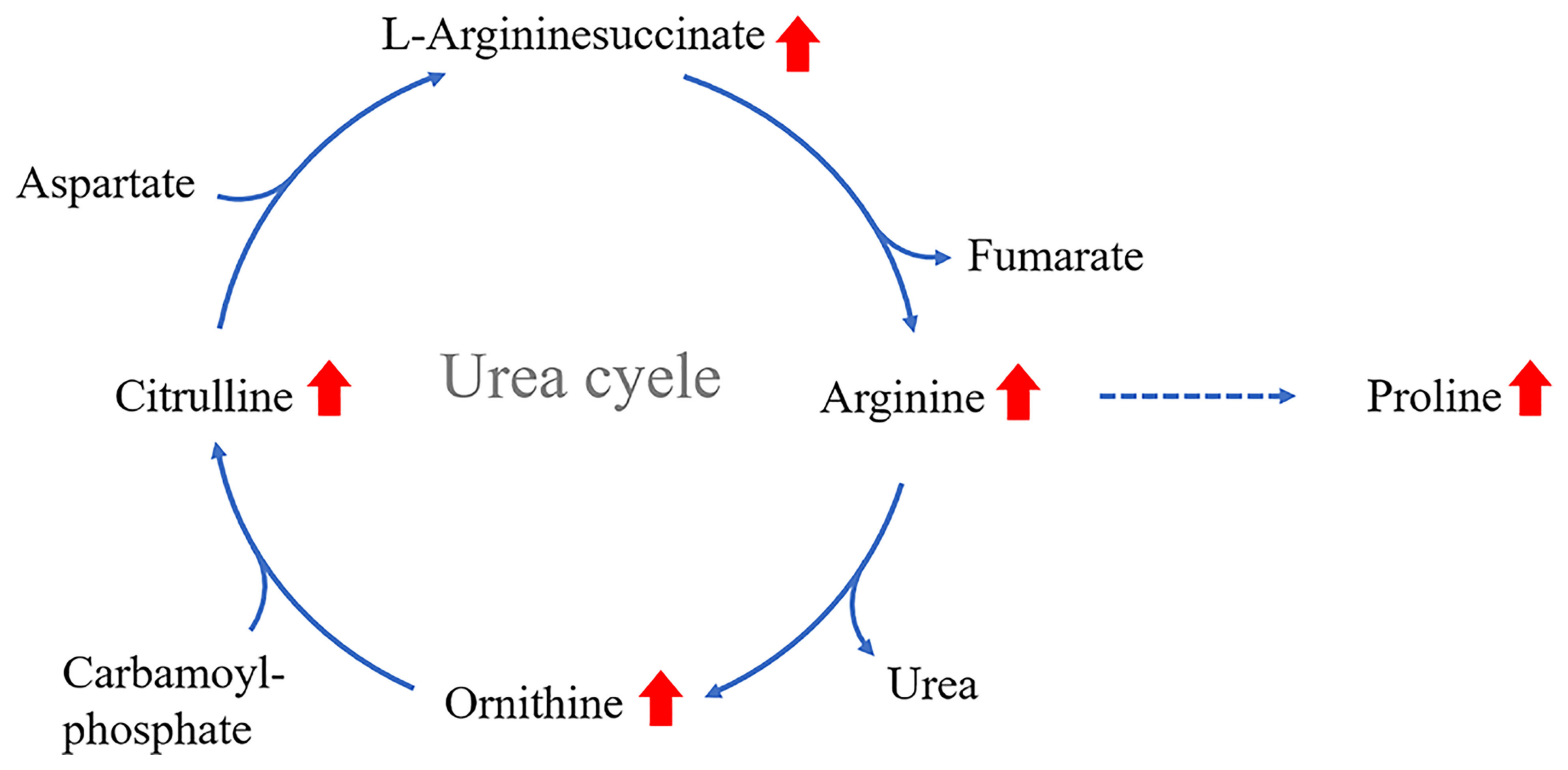
Increased levels of proline, ornithine and arginine in the vitreous humor of PDR patients; arginine levels are elevated in the serum of severe DR patients; citrulline levels are elevated in the aqueous humor of DR patients.

The urea cycle is a part of the arginine biosynthesis pathway, and the arginase enzyme can cleave arginine to generate urea and ornithine. Ornithine can be converted into citrulline, and then citrulline is produced through a series of reactions to arginine (101). The metabolites of the urea cycle seem to have some association with DR. The metabolites of the urea cycle seem to have some association with DR.The levels of ornithine (85, 97, 102), arginine (62, 90, 102), citrulline (85, 90, 102), proline (86), and argininosuccinate (102) were significantly elevated in DR patients (**Figure 2**) (73). The above content expands our understanding of the pathogenesis of DR. The changes in the metabolites of the urea cycle, especially arginine, are significantly associated with DR.

**Figure 3:**
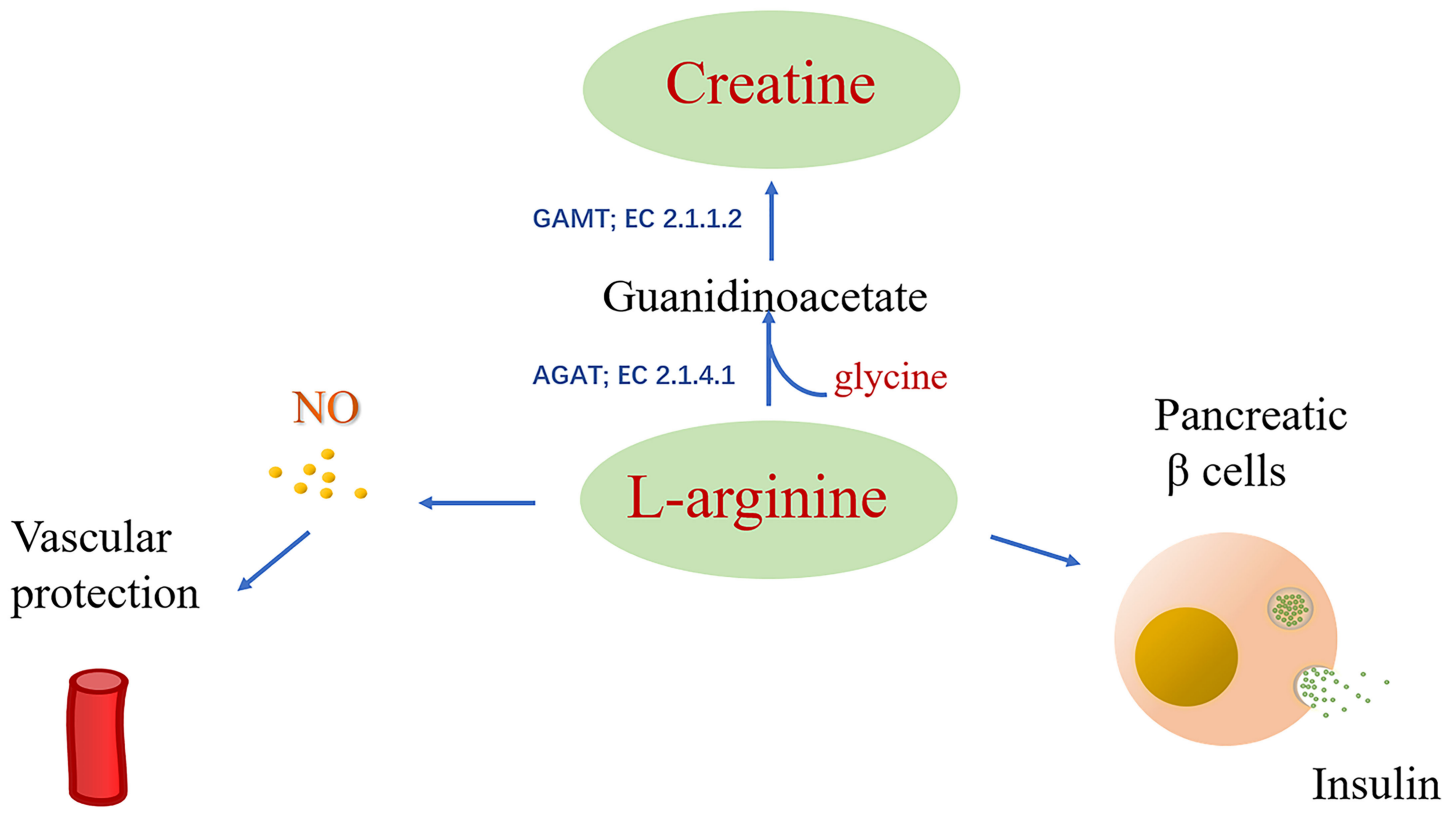
Arginine is catalyzed by the substrate of nitric oxide synthase (NOS) to produce NO, and arginine can induce the release of insulin from pancreatic β cells.

Arginine is involved in many biological processes and is also the substrate of nitric oxide synthase (NOS) and arginase, producing nitric oxide (NO) and urea, respectively (103). NO is a vasodilator that exerts a significant influence on vascular endothelial health, while arginine induces the release of insulin in pancreatic β cells (**Figure 3**) (104). In addition, animal experiments using DR mouse models and bovine retinal endothelial cells cultivated by high glucose revealed the role of arginine metabolism as a mediator for DR (105, 106).

### Arginine-Creatine Metabolic Pathway May Be a New Therapeutic Strategy for DR

Meanwhile, another biomarker that caught our attention, creatine, a product of arginine metabolism. Unlike the elevated levels of arginine, creatine levels were significantly lower in patients with DR (84, 85). Thereby, we put forward a hypothesis that the reduced conversion of arginine to creatine leads to metabolic changes in DR patients with increased arginine levels and decreased creatine levels. Callback of this metabolic change, may be a new treatment strategy for DR. There is no strong evidence for this hypothesis, but there is substantial research supporting the positive effects of creatine supplementation on DR.

Creatine can be either be synthesized endogenously within the body or extrinsically derived from foods like meat, fish, etc. (24). There are two steps in creatine biosynthesis. The first step is to catalyze arginine and glycine with L-arginine glycine amidinotransferase (AGAT; EC 2.1.4.1) to produce ornithine and guanidinoacetate (GAA). This step mainly occurs in the kidney and is mostly distributed in the mitochondrial intermembrane space (107). The second step is the methylation of GAA in the amidino group for producing Cr through the action of S-adenosyl-l-methionine: N-guanidinoacetate methyltransferase (GAMT; EC 2.1.1.2) (108), the liver is possible to be the principal organ contributing this reaction (109, 110). Approximately two-thirds of Cr is phosphorylated to form PCr, a key agents of cellular energy regeneration (111, 112). Cr, PCr, and creatine kinase (CK) isoenzymes are responsible for maintaining the ATP pool (25). This is critical for some organs with high energy demands, like retina, skeletal or cardiac muscle, retina, spermatozoa, and brain (113).

AGAT is the rate-limiting enzyme in creatine biosynthesis, simultaneous reduction in mRNA content, enzyme levels, and AGAT enzyme activity when endogenous sources or dietary Cr supplementation (114). This feedback inhibition of AGAT by Cr is most pronounced in the kidney and pancreas, which are the major tissues for GAA production (115). Research shows that ingestion of creatine supplements reduces the rate of creatine biosynthesis (116).GAA, catalyzed by GAMT to generate creatine, is an important intermediate in creatine biosynthesis. Deficiency of GAMT will cause GAA accumulation and lead to axonal hypersprouting and apoptosis (117). There are no reports of abnormal GAA levels in DR patients.

Studies have shown that creatine supplementation can help improve hyperglycemia (34) and improve glycemic control in patients with type 2 diabetes (118). In mice, lower creatine levels could be ascribed to the vascular proliferation of the retina under the OIR model (p=0.027) with the use of retinal metabolomics. Moreover, it was seen that this vascular proliferation could be reversed after the administration of oral creatine *via* anti-VEGF (84). Tomita et al. found that a decrease in creatine was accompanied by an increase in glycine levels in OIR mice, this results consistent with the vitreous humor of PDR patients (84). Glycine is involved in the biosynthesis of creatine, the amidine group of arginine is transferred to glycine to generate ornithine and GAA, and then GAA is catalyzed by GAMT to generate creatine. Increased glycine appears to be protective for DR, and glycine has proven anti-glycation and anti-diabetic properties (119, 120). Moreover, glycine significantly upregulated the mRNA expression of PEDF (an angiogenesis inhibitor) (121). However, in the study by Tomita et al., arginine was not significantly different in the vitreous humor of PDR patients and the retina of OIR mice. In previous studies, arginine was reported to be significantly elevated in plasma and vitreous humour (62, 90, 102).

Mitochondria are the primary site of production ATP and the main source of cellular energy. The number of mitochondria in a cell depends on its energy demand (122). Mitochondrial dysfunction due to overproduced of ROS in hyperglycemic states (122, 123), and make a major impact on tissues with high energy demands, such as the retina (111). Study shows persistent hyperglycemia leads to reduced mitochondrial respiration (124), Cr-Pcr system is essential for energy-demanding tissues and cells due to the maintenance of adequate ATP pools (111).

Another study showed that creatine enhanced the functional capillary density in skin and recruitment in post-occlusive reactive hyperemia (35, 125). The author speculates that creatine may help increase the bioavailability of epoxyeicosatrienoic acid (EET), thereby improving endothelium-derived hyperpolarizing factor (EDHF) stimulation and microvascular dilation (125). Apart from this, the potential therapeutic effect of creatine on the nervous system also deserves attention. It has been reported that creatine protects against neurotoxicity and oxidative stress (30, 31). Oxidative stress is one of the biggest risk factors for diabetic retinopathy. An animal experiment demonstrated that creatine has a significant antioxidant effect and indicated that creatine supplementation may become a treatment strategy for neurodegenerative diseases caused by oxidative stress (34, 126). Besides, creatine administration significantly attenuated abnormal glucose tolerance, and is considered to delayed the onset of diabetes (34). Studies have shown that creatine exhibits resistance to oxidation, which is effective in protecting mtDNAs from oxidative stress-elicited cytotoxicity (127, 128). Suggestively, creatine could provide a way for the effective management of diseases involving oxidative stress (126–128).

Synthesis of creatine yields homocysteine as a byproduct, which is an amino acid that contains sulfhydryl groups. S-adenosylmethionine (SAM) is demethylated to generate creatine as well as S-adenosyl homocysteine (SAH). SAH hydrolase (SAHH) enzyme then hydrolyzes SAH to Hcy. A correlation has been reported between the increase in Hcy expression and an aggravated risk for diverse DR, including blood retinal barrier dysfunction, inflammation, and mitochondria dysfunction (129–131). Replenishment of creatine has been demonstrated to save the SAM input (132–134) given about 40–70% expenditure of entire methyl groups by the creatine synthesis (134), which can diminish the Hcy formation (133) and may help reduce the possibility of developing DR.

**Figure 4:**
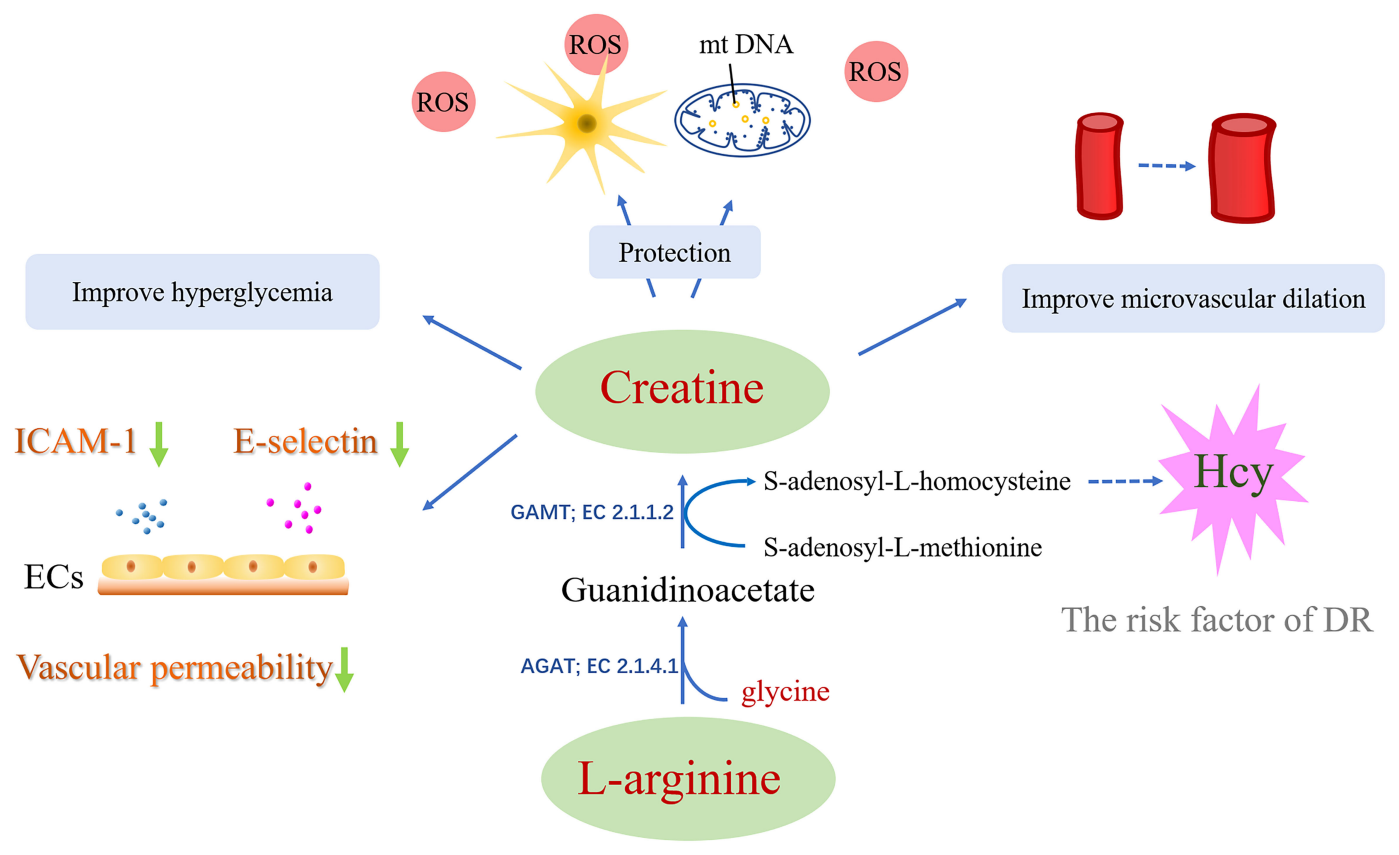
The approach of creatine in treating diabetic retinopathy: i) Creatine has the potential to act as an anti-inflammatory aid and provide vascular protection. ii) Creatine has a significant antioxidant effect and protects mtDNA and nerve cells from cytotoxicity induced by oxidative stress. iii) Creatine may help increase the bioavailability of epoxyeicosatrienoic acid, thereby improving microvascular dilation. iv) Creatine may reduce the formation of Hcy. v) Creatine supplementation can help improve hyperglycemia.

In addition, creatine can reduce acute inflammation induced by carrageenan, whose action is identical to that of butazepine, a non-steroidal anti-inflammatory drug (27). Research done by Nomura et al. on pulmonary endothelial cells (ECs) revealed that after the administration of 0.5 mM creatine, the endothelial cell (EC) expressions of E-selectin and Intercellular Adhesion Molecule-1 were suppressed. Moreover, the serotonin-and H2O2-elicited permeability of endothelium was also prominently reduced upon creatine (5 mM) replenishment. These observations suggested that the administration of creatine makes the membranes more stable, and the ECs less leaky (28). Associations between DR and increased intercellular cell adhesion molecule-1 (ICAM-1), E-selectin expressions, and enhanced permeability “leakiness” of the endothelium have been reported several times (135). It shows that creatine has the potential to act as a protector of the vascular system and as an inflammation inhibitor (**Figure 4**).

## Conclusion

In recent years, researchers have identified many potential DR biomarkers, which are not yet used for clinical diagnosis. Further research is required to clarify their molecular mechanisms in DR. In this review, we have discussed the known biomarkers of diabetic retinopathy, which can help in predicting and preventing DR in the future. Furthermore, we suggest that the arginine-creatine metabolic pathway may be a new strategy for the treatment of diabetic retinopathy.

## Author Contributions

YS, LK, A-HZ, YH, HS, and G-LY analyzed the data. YS wrote the paper. A-HZ and X-JW revised the paper. All the authors read and approved the final manuscript.

## Funding

This work was supported by grants from the Key Program of Natural Science Foundation of State (Grant No. 81973745, 81830110), Natural Science Foundation of Heilongjiang Province (YQ2019H030), Heilongjiang Touyan Innovation Team Program.

## Conflict of Interest

The authors declare that the research was conducted in the absence of any commercial or financial relationships that could be construed as a potential conflict of interest.

## Publisher’s Note

All claims expressed in this article are solely those of the authors and do not necessarily represent those of their affiliated organizations, or those of the publisher, the editors and the reviewers. Any product that may be evaluated in this article, or claim that may be made by its manufacturer, is not guaranteed or endorsed by the publisher.

## Abbreviation

DR, Diabetic retinopathy; T2DM, type 2 diabetes mellitus; PKC, protein kinase C; Cr, creatine; PCr, phosphocreatine; CK, creatine kinase; NMR, nuclear magnetic resonance; MS, mass spectrometry; LC, liquid chromatography; GC, gas chromatography; HILIC-MS, hydrophilic interaction chromatography-mass spectrometry; FIA–MS, Flow-injection analysis-mass spectrometry; CE, capillary electrophoresis; HPLC, high-performance liquid chromatography; UPLC, ultra-high performance liquid chromatography; CE, Capillary electrophoresis; PDR, proliferative diabetic retinopathy; OIR, oxygen induced ischemic retinopathy; GC-MS, gas chromatography mass spectrometry; UPLC-MS, ultra-performance liquid chromatography-mass spectrometry; TCA, tricarboxylic acid cycle; LC-MS, liquid chromatography-mass spectrometry; HILIC-MS Hydrophilic interaction chromatography-mass spectrometry; UPLC-Q-Axis Orbiter-MS, ultra-performance liquid chromatography-quadrupole-Exactive Orbitrap-mass spectrometry; IDO, indolamine-2,3-dioxygenase; UACR, albumin/creatinine; KEGG, Kyoto Encyclopedia of Genes and Genomes; NOS, nitric oxide synthase; NO, nitric oxide; EDHF, endothelium-derived hyperpolarizing factor; GAA, guanidinoacetate; Hcy, homocysteine; ECs, endothelial cells; EC, endothelial cell; LPC, Lysophosphatidylcholine; ICAM-1, increased intercellular cell adhesion molecule-1.
